# Accuracy of a flash glucose monitoring system in dogs with diabetic ketoacidosis

**DOI:** 10.1111/jvim.15657

**Published:** 2019-11-14

**Authors:** Eleonora Malerba, Chiara Cattani, Francesca Del Baldo, Gaia Carotenuto, Sara Corradini, Stefania Golinelli, Ignazio Drudi, Federico Fracassi

**Affiliations:** ^1^ Department of Veterinary Medical Sciences University of Bologna, Ozzano dell'Emilia Bologna Italy; ^2^ Department of Statistical Sciences University of Bologna Bologna Italy

**Keywords:** canine, freestyle libre, interstitial glucose, noninvasive glucose measurement

## Abstract

**Background:**

A factory‐calibrated flash glucose monitoring system (FGMS; FreeStyle Libre) recently was evaluated in dogs with uncomplicated diabetes mellitus. It is not known if this system is reliable during diabetic ketoacidosis (DKA).

**Objectives:**

To assess the performance of the FGMS in dogs with DKA and to determine the effect of severity of ketosis and acidosis, lactate concentration, body condition score (BCS), and time wearing the sensor on the accuracy of the device.

**Animals:**

Fourteen client‐owned dogs with DKA.

**Methods:**

The interstitial glucose (IG) measurements were compared with blood glucose (BG) measurements obtained using a validated portable glucometer. The influence of changes in metabolic variables (β‐hydroxybutyrate, pH, bicarbonate, and lactate) and the effect of BCS and time wearing on sensor performance were evaluated. Accuracy was determined by fulfillment of ISO15197:2013 criteria.

**Results:**

Metabolic variables, BCS, and time wearing were not associated with the accuracy of the sensor. Good agreement between IG measurements and BG was obtained both before and after DKA resolution (*r* = .88 and *r* = .93, respectively). Analytical accuracy was not achieved, whereas clinical accuracy was demonstrated with 100% and 99.6% of results in zones A + B of the Parkes consensus error grid analysis before and after DKA resolution, respectively.

**Conclusions and Clinical Importance:**

Changes in metabolic variables, BCS, and time wearing do not seem to affect agreement between IG and BG. Despite not fulfilling the ISO requirements, the FGMS provides clinically accurate estimates of BG in dogs with DKA.

**Abbreviations**
BCS: body condition score
BG: blood glucose
BHB: beta‐hydroxybutyrate
CGMS: continuous glucose monitoring system
DKA: diabetic ketoacidosis
EGA: error grid analysis
FGMS: flash glucose monitoring system
IG: interstitial glucose
MAD: mean absolute difference
MARD: mean absolute relative difference
mARD: median absolute relative difference
MRD: mean relative difference
PBGM: portable blood glucose meter

## 1 INTRODUCTION

Diabetic ketoacidosis (DKA) is the most common life‐threatening complication of diabetes mellitus, involving extreme alterations of metabolic variables. The syndrome is characterized by a biochemical triad of hyperglycemia, ketosis, and acidosis.^1^, ^2^, ^3^, ^4^, ^5^ Treatment of DKA involves IV fluid resuscitation, correction of acid–base and electrolyte derangements, insulin administration, as well as identification and treatment of any concurrent illness.^5^ Insulin treatment aims to support cellular glucose uptake, decrease hepatic glucose production, interrupt the process of ketogenesis, and promote ketone metabolism and clearance.^6^, ^7^ Frequent glucose monitoring is necessary during treatment as a result of insulin administration, glucose supplementation, and compromised homeostatic mechanisms that are characteristic of ketoacidotic patients. Currently, hospitalized ketoacidotic patients usually are monitored by measuring blood glucose (BG) concentration using a portable blood glucose meter (PBGM). The main limitation of this device is the need for frequent phlebotomies that can lead to iatrogenic anemia (an important cause for increased transfusion requirement and longer duration of hospitalization, especially in small breed dogs and cats),^8^, ^9^ or alternatively placement of a second or central catheter for blood sampling, increasing the risk of catheter‐related complications, including infection and phlebitis.^10^, ^11^, ^12^ Moreover, such BG monitoring methods allow only intermittent assessment of BG concentration (usually every 1‐2 hours), limiting the amount of information available on which to base treatment decisions. Finally, these methods can increase patient stress, owner expense, and workload of nursing staff and clinicians. For these reasons, research is being directed toward less invasive methods to monitor BG concentrations continuously in patients with DKA.

In the past 2 decades, there has been growing interest in devices measuring interstitial glucose (IG) concentration, which has been shown to reflect circulating BG concentrations. Several studies have evaluated their accuracy in humans has well as horses, cows, dogs, cats, rats, and rabbits.^13^, ^14^, ^15^, ^16^, ^17^, ^18^, ^19^, ^20^, ^21^

The first‐generation systems offered only retrospective analysis of glucose concentrations after disconnecting the sensor and uploading the data (continuous glucose monitoring system, CGMS), whereas second‐generation instruments measured and displayed the data immediately, allowing direct intervention (real‐time CGMS).^22^ However, the need for blood collection was not eliminated completely, because these monitoring systems must be calibrated 2 to 3 times per day, requiring BG measurement using capillary or venous blood sampling.^22^, ^23^ A novel factory‐calibrated flash glucose monitoring system (FGMS, FreeStyle Libre, Abbott, UK) has been licensed for use in people (CE mark, August 2014). The system consists of a small, round, disposable, water‐resistant sensor, which continuously measures glucose in the interstitial fluid through a small (5 mm long × 0.4 mm wide) filament inserted SC. The FGMS generates information every minute, and the readings are automatically stored in 15‐minute intervals for up to 14 days. Interstitial glucose concentrations are displayed when the sensor is wirelessly scanned (or “flashed”) with a reader device on demand. The reader device then will display the past 8 hours of glucose information, including current glucose, a trend graph, and a trend arrow that indicates the direction of the patient's current glucose concentration with respect to the previous results. The FGMS recently has been evaluated in diabetic dogs without DKA,^21^ but not in dogs with DKA, which typically have substantial metabolic alterations that could affect the accuracy of the device.

Our aims were to assess the performance of the FGMS in dogs during DKA and after its resolution, comparing IG measurements with BG concentrations obtained with a PBGM, and to determine the effect of BCS, lactate concentration, severity of ketosis, and acidosis on the accuracy of the device.

## 2 MATERIALS AND METHODS

### 2.1 Dogs

Client‐owned dogs admitted to the University Veterinary Teaching Hospital of Bologna between April 2015 and July 2017 with naturally occurring DKA were enrolled in the study. The diagnosis of DKA was based on the presence of at least 2 clinical signs consistent with DKA (eg, polyuria, polydipsia, anorexia, severe lethargy, vomiting, dehydration), BG concentration >250 mg/dL, blood β‐hydroxybutyrate (BHB) concentration >3.8 mmol/L,^24^ and venous pH <7.3 or bicarbonate <15 mEq/L. The dogs were treated according to a modified previously published protocol,^6^ using IV continuous rate infusion of regular insulin (Humulin R, Ely Lilly and Co, Indianapolis, Indiana). Diabetic ketoacidosis was considered resolved when BHB was ≤1.0 mmol/L and when venous pH was ≥7.3, bicarbonate was ≥15 mEq/L or both.

The Scientific Ethics Committee of the University of Bologna approved this study, and owners signed a written informed consent form before enrollment.

### 2.2 Data collection

**Figure 1:**
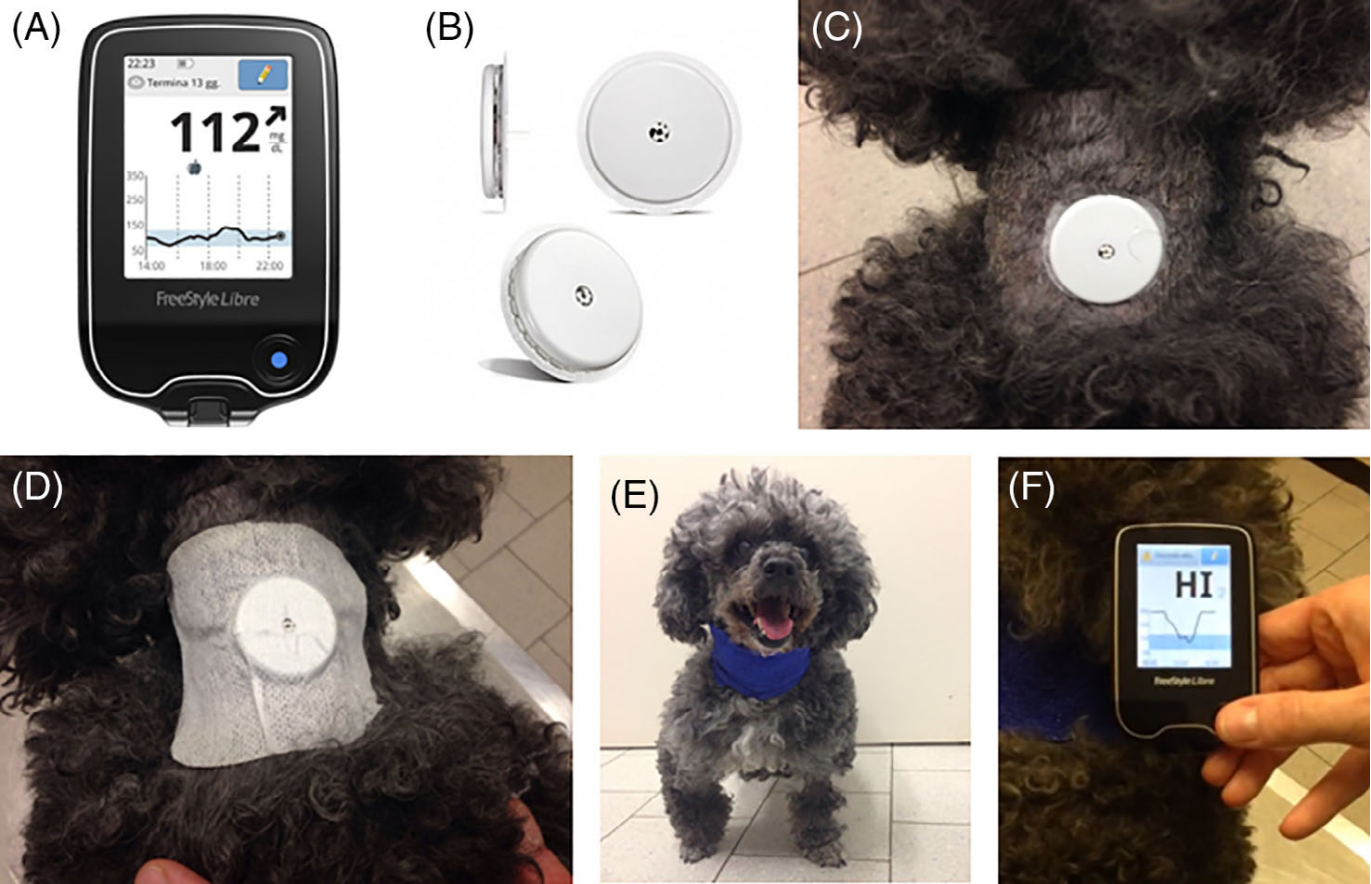
FreeStyle Libre is composed of the reader (A) and the sensor (B), which is placed on the dorsal part of the neck of the dog (C), secured by an additional tape (D) and a bandage applied around the neck (E). The sensor has to be scanned by the reader, which instantaneously shows the interstitial glucose value (F). The reader shows “HI” and “LO” when the interstitial glucose concentration is ≥500 mg/dL and ≤20 mg/dL, respectively

In dogs admitted during working hours, the FGMS was applied as soon as the diagnosis of DKA was confirmed; in dogs admitted out‐of‐hours, application was postponed until the next morning. The sensor was placed on a clipped and clean area of the dorsal part of the neck, and adherence to the skin was further ensured by additional tape (Pic Solution Soffix Stretch, Pikdare Srl, Como, Italy) and bandage (Vetrap, 3M Italia Srl, Milano, Italy) applied around the neck (Figure 1).^21^ The sensor has a 1‐hour period of initialization. The detection limits of the sensor are between 20 and 500 mg/dL; when the IG concentration is ≤20 mg/dL and ≥500 mg/dL, the reader shows “LO” and “HI,” respectively. The IG measurements were compared with BG concentrations obtained within 10 seconds by a PBGM (Optium Xceed, Abbott, UK), validated for use in dogs.^25^ Venous or capillary BG concentrations were measured every 1‐2 hours from admission to the resolution of DKA, and then less frequently, at the clinician's discretion according to the patient's condition, until discharge.

Body condition score (BCS) was recorded at admission using a previously described 9‐point scoring system (World Small Animal Veterinary Association Global Nutrition Committee, BCS chart). The patient's metabolic status (pH and bicarbonate) and lactate concentration (marker of tissue perfusion) were assessed by blood gas analysis, performed using a blood gas analyzer (ABL 800 Flex, Radiometer Medical ApS, Brønshøj, Denmark), every 8‐12 hours until DKA was resolved and then 24 hours later to confirm that the patient's metabolic balance had been maintained. The degree of ketosis was quantified every 4 hours by measuring blood BHB using the same PBGM (using ketone test strips), previously validated for dogs.^24^

At the end of wearing period, which coincided with discharge, the sites of sensor application on all dogs were judged subjectively for the presence of erythema or other adverse events by the same clinician (C.C.).

### 2.3 Statistical analysis

The influence of changes in metabolic variables (β‐hydroxybutyrate, pH, bicarbonate, and lactate) and the effect of BCS and time wearing on sensor performance were evaluated to investigate whether specific patient variables influenced the accuracy of the device during the resolution of DKA. For this purpose, a mixed model was used to investigate the significance of covariates on IG measurements. The mixed model was conducted imposing random effects on subjects and fixed effects on BG and covariates. This was performed for all observations (DKA all samples), for observations obtained before DKA resolution as well as observations after DKA resolution. Because the number of BG and IG measurements varied among covariates, separate mixed models were performed for each variable. The basic model was defined as:

IG=intercept+BG+XX+random effectonsubject+error term,

where *XX* represents one of the covariates in the study (β‐hydroxybutyrate, pH, bicarbonate, lactate, BCS, or time wearing).

The correlations between IG measured by the FGMS and BG measured by the PBGM for all samples and for samples obtained both before and after DKA resolution were evaluated using repeated measures correlation (*rmcorr*).

Statistical analysis was performed using commercially available software (R Core Team, 2019. R: A language and environment for statistical computing. R foundation for Statistical Computing, Vienna, Austria).

### 2.4 Accuracy of the FGMS

Analytical and clinical accuracy during DKA and after its resolution was evaluated by comparing the results of the PBGM measurements and those obtained using the FGMS, using the ISO 15197:2013 criteria (BSI Standards Publication, in vitro diagnostic test system—Requirements for BG monitoring system for self‐testing in managing diabetes mellitus; EN ISO 15197:2013).

Analytical accuracy was determined by calculating the mean absolute relative difference (MARD), median absolute relative difference (mARD), mean relative difference (MRD), and mean absolute difference (MAD). All these are measures of the average difference between sensor and reference results. Mean absolute relative difference and mARD measure the size but not the direction (higher or lower) of the differences compared with the reference (absolute) as a percentage of the reference value (relative). Mean absolute difference is similar, but just reports the size of the difference (it is not reported as a percentage), and is commonly used to assess accuracy at low BG concentrations (< 100 mg/dL). Mean relative difference measures the size and direction of the difference compared with the reference as a percentage of the reference value.^26^ Mean absolute relative difference traditionally has been used to assess the accuracy of CGMSs, representing it as a single numeric value.^27^ Mean absolute relative difference or mARD should be <14%; a value >18% is considered to represent poor accuracy.^28^

Second, analytical accuracy was estimated based on ISO 15197:2013 criteria, which state that at least 95% of results must be within ±15 mg/dL of the BG concentration for BG concentration <100 mg/dL and within ±15% of the BG concentration for BG concentration ≥100 mg/dL.

Clinical accuracy was evaluated using Parkes consensus error grid analysis (EGA) for type 1 DM, which categorizes errors in BG measurement in terms of clinical risk.^29^ In this analysis, a scatter plot is generated of the estimated BG concentrations (in our case, IG measurements obtained by the FGMS, y‐axis) versus measured BG concentrations (glycemia obtained by the PBGM, x‐axis). This plot is divided into 5 zones (A‐E), based on the assumption that the clinical goal is to maintain BG concentration between 70 and 180 mg/dL. The 5 zones are defined as follows: (A) no effect on clinical action; (B) altered clinical action unlikely to affect outcome; (C) altered clinical action likely to affect clinical outcome; (D) altered clinical action could have substantial medical risk; and (E) altered clinical action could have dangerous consequences. Based on the ISO 15197:2013 criteria, 99% of the measured glucose results should fall within zones A and B of Parkes EGA.

## 3 RESULTS

Fourteen dogs were included in the study; of those, 2 had BCS 3, 5 had BCS 4, 3 had BCS 5, 2 had BCS 7, 1 had BCS 8, and 1 had BCS 9. The application of the FGMS was carried out (median [min‐max]) 3 (1‐13) hours after admission. The application appeared to be painless, easy to perform, and was well tolerated by all dogs. In all subjects, the sensor started reading the IG concentrations after 60 minutes of application, as reported by the manufacturer. Data were collected from each patient for a minimum of 3 days and up to 14 days (median, 5.5 days). No relevant adverse events were recorded during the use of the FGMS; only in 1 dog was mild erythema noted at the site of application of the sensor at the end of the wearing period, which spontaneously resolved within the next 24 hours.

A total of 485 paired glucose measurements were available for analysis, of which 229 were obtained during DKA and 256 after its resolution. Before DKA resolution, the median BG concentration measured by the PBGM and the median IG concentration obtained by the FGMS were 240 mg/dL (range, 76‐499 mg/dL) and 236 mg/dL (range, 54‐500 mg/dL), respectively; after DKA resolution, the median BG concentration measured by the PBGM and the median IG concentration obtained by the FGMS were 274.5 mg/dL (range, 57‐498 mg/dL) and 261.5 mg/dL (range, 47‐492 mg/dL), respectively.

From admission until 24 hours after DKA resolution, 53 pH and bicarbonate concentrations and 52 lactate concentrations were collected. Beta‐hydroxybutyrate was measured up to discharge, resulting in 135 results. The median pH and bicarbonate concentrations were 7.27 (range, 7.03‐7.40) and 14.9 mmol/L (range, 7.8‐23.3 mmol/L), respectively; the median lactate concentration was 1.2 mmol/L (range, 0.5‐2.8 mmol/L). The median BHB concentration throughout hospitalization was 1.7 mmol/L (range, 0.1‐7.5 mmol/L). The results of mixed models for each covariate showed that none of the covariates influenced the relationship between IG and BG (Table 1). At this point, we adopted a mixed model approach but imposing random effect on patients. Good correlations between IG and BG measurements were obtained using *rmcorr* for all samples (*r* = .91) as well as before (*r* = .88) and after DKA resolution (*r* = .93; Table 2).

**Table 1:** Number of observations (n) and significance of *t*‐test estimated by mixed models for each covariate in the whole set of observations (*DKA all samples*) and in observations obtained *before DKA resolution* and *after DKA resolution*

	DKA all samples		Before DKA resolution		After DKA resolution	
Covariate	n	Significance	n	Significance	n	Significance
β‐hydroxybutyrate	135	.804	66	.400	69	.661
pH	53	.407	35	.637	18	.569
Bicarbonate	53	.156	35	.904	18	.603
Lactate	52	.172	34	.930	18	.064
BCS 3	58	.561	26	.611	32	.411
BCS 4	184	.648	76	.738	108	.708
BCS 5	59	.806	44	.956	15	.866
BCS 7	85	.436	41	.486	44	.576
BCS 8	71	.199	26	.329	45	.319
BCS 9	28	.756	16	.886	12	.876
Time	485	.349	229	.160	256	.905

Abbreviations: BCS, body condition score; DKA, diabetic ketoacidosis.

**Table 2:** Measurement model: IG = intercept + BG + random effect on subject + error term

	n	Estimate	Standard Error	*t* value	Significance	*r*
DKA all samples						
Intercept	485	25.493	13.955	1.830	.068	.92
BG	485	0.931	0.019	48.290	0	
Before DKA resolution						
Intercept	229	45.696	14.331	3.189	.002	.88
BG	229	0.849	0.032	26.898	0	
After DKA resolution						
Intercept	256	13.965	14.032	1.000	.318	.93
BG	256	0.976	0.025	38.360	0	

*Note*: The *r* values, estimated using *rmcorr*, indicate the correlations between IG and BG measurements in the whole set of observations (*DKA all samples*) and in observations obtained *before DKA resolution* and *after DKA resolution*.

Abbreviations: BG, blood glucose; DKA, diabetic ketoacidosis; IG, interstitial glucose.

**Figure 2:**
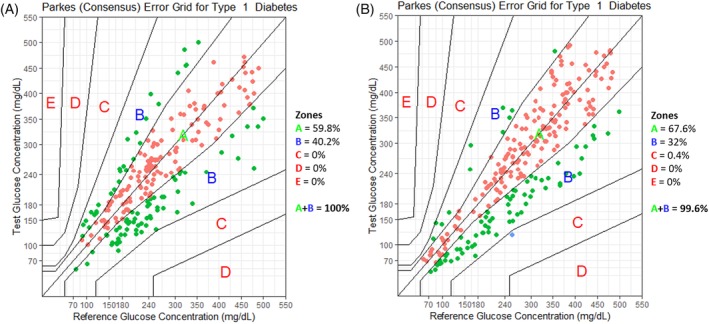
Parkes consensus error grid analysis (EGA) representation with the percentage of values within different zones before diabetic ketoacidosis (DKA) resolution (A) and after DKA resolution (B). The reference glucose values (blood glucose obtained by a portable glucometer), on the x‐axis, are plotted against the interstitial glucose measurements obtained by the flash glucose monitoring system, on the y‐axis. The different zones designate the magnitude of risk: no effect on clinical action (zone A), altered clinical action—little or no effect on the clinical outcome (zone B), altered clinical action—likely to affect the clinical outcome (zone C), altered clinical action—could have a significant medical risk (zone D), and altered clinical action—could have dangerous consequences (zone E). ISO 15197:2013 requires that 99% of the values fall within zones A + B for a device to be considered accurate

Before DKA resolution, in the low glucose range (BG < 100 mg/dL, *n* = 5) MAD was 34.0 mg/dL; in the higher glucose range (BG ≥100 mg/dL, *n* = 224) MARD was 19.7%, mARD was 18.2%, and MRD was −3.5%. After DKA resolution, in the low glucose range (*n* = 21) MAD was 22.7 mg/dL; in the higher glucose range (*n* = 235) MARD was 17.2%, mARD was 13.7%, and MRD was −6.0%. Before DKA resolution, the percentages of values within ±15 mg/dL of the BG concentration for BG concentrations <100 mg/dL and within ±15% of the BG concentration for BG concentrations ≥100 mg/dL were 0% (0/5) and 43.8% (98/224), respectively. After DKA resolution, the percentages of results within ±15 mg/dL of the BG concentration for BG concentrations <100 mg/dL and within ±15% of the BG concentration for BG concentrations ≥100 mg/dL were 42.9% (9/21) and 53.6% (126/235), respectively (Table 3). Evaluation of data using the Parkes consensus EGA showed that 100% of the FGMS results fell in zones A and B, deemed clinically acceptable, before DKA resolution; only 1 of the FGMS measurements obtained after DKA resolution fell in zone C (0.4%), and all of the remaining results fell in zones A and B (99.6%; Figure 2).

**Table 3:** Results of Freestyle's analytical accuracy in the low (BG < 100 mg/dL) and high glucose range (BG ≥ 100 mg/dL) before and after DKA resolution

	Before DKA resolution	After DKA resolution
Low glucose range (BG <100 mg/dL)		
n	5	21
MAD (mg/dL)	34.0	22.7
Percent of values within ±15 mg/dL of the BG value	0% (0/5)	42.9% (9/21)
High glucose range (BG ≥100 mg/dL)		
n	224	235
MARD (%)	19.7	17.2
mARD (%)	18.2	13.7
MRD (%)	−3.5	−6.0
Percent of values within ±15% of the BG value	43.8% (98/224)	53.6% (126/235)

Abbreviations: BG, blood glucose; DKA, diabetic ketoacidosis; MAD, mean absolute difference; MARD, mean absolute relative difference; mARD, median absolute relative difference; MRD, mean relative difference.

**Figure 3:**
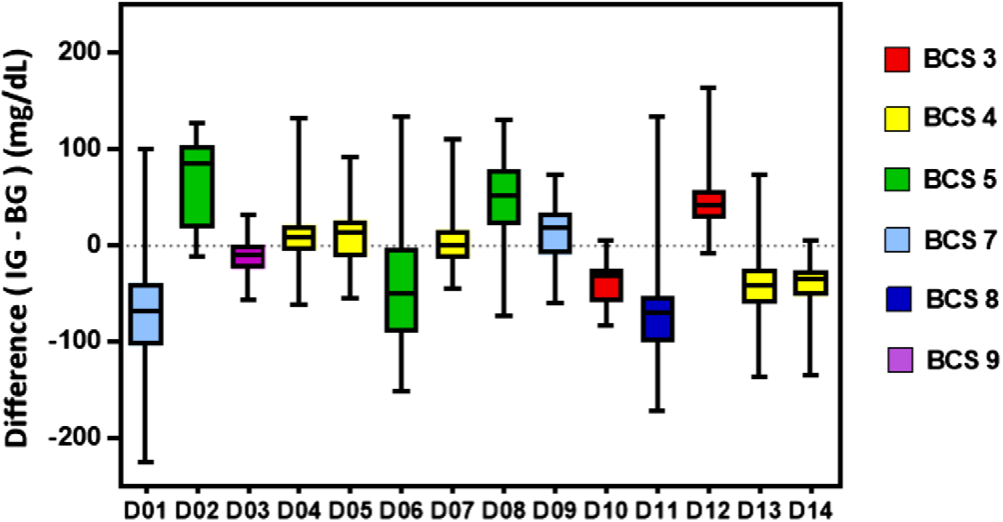
Inter‐patient variability (D = dog). Each patient is represented on the x‐axis with a box and whisker plot. The y‐axis represents the relative difference defined as IG − BG. BG, blood glucose; IG, interstitial glucose

Figure 3 shows the distributions of the differences between IG measurements obtained with the FGMS and BG concentrations obtained with the PBGM for each patient. A significant inter‐patient variability in the accuracy of the device was observed (Kruskal‐Wallis test, *P* < .0001), suggesting that in some patients the device was more accurate than in others.

## 4 DISCUSSION

Our study is the first to evaluate the clinical accuracy and performance of the FGMS in dogs with DKA. Although it does not fulfill the analytical ISO 2013 accuracy requirements, the FGMS demonstrated acceptable clinical accuracy to be used as an IG monitoring tool in these patients.

Diabetic ketoacidosis is an endocrine emergency that represents a very difficult metabolic therapeutic challenge in veterinary medicine. Patients with DKA require intense supervision and monitoring because of the potential for complications arising from treatment. Examples of complications include hypoglycemia, hypokalemia, hypophosphatemia, and cerebral edema.^2^, ^4^, ^5^, ^30^, ^31^, ^32^ These complications usually result from overly aggressive treatment, inadequate animal monitoring, and failure to reevaluate biochemical variables in a timely manner.^5^ During the past decades, the mortality rate has decreased from 26% to 30%^1^, ^2^, ^6^ to approximately 5% to 7%,^4^, ^5^ and death usually has been attributed to underlying medical disorders (eg, pancreatitis) that precipitated the DKA, client financial constraints, or both rather than to the metabolic complications of ketoacidosis. These improvements in outcome could be a result of increased knowledge about the pathophysiology of DKA, but also to the application of new treatment and monitoring techniques. Glycemic monitoring is a cornerstone for the management of DKA, and currently it typically is performed using PBGMs. The main limitations of these devices include the cost of test strips and the requirement for repeated capillary or venous blood sampling, which can be a source of stress and pain in some patients. Moreover, PBGMs only provide single snapshots of glycemia so it is possible that rapid changes in BG concentrations can be missed and not factored into treatment decisions. In ketoacidotic patients, accurate and frequent glucose monitoring is the best way to avoid a rapid decrease in the BG concentration, which could result in cerebral edema and hypoglycemia, and to allow correct management of insulin treatment. The FreeStyle Libre is unique among existing IG monitoring technologies in that the wired enzyme factory‐calibrated sensor has a wearing time of up to 14 days without additional calibration, which represents a potential advantage. Moreover, the FGMS provides IG results across a wide range of BG concentrations, between 20 and 500 mg/dL, and numerous readings during a 24‐hour period that can be used to evaluate glycemic patterns and trends because the hand‐held reader displays the previous 8‐hour history. This ability to foresee and avoid impending hyperglycemic and hypoglycemic events in critically ill ketoacidotic patients potentially could improve both morbidity and mortality in patients. The maximum upper range of 500 mg/dL is appropriate for dogs with DKA, in which glycemic control (BG concentration around 250 mg/dL) usually is a major goal of treatment.

In human medicine, glucose management in intensive care unit patients, with and without diabetes, has been a matter of debate for almost 2 decades. A recent consensus stated that, compared to intermittent monitoring systems, continuous glucose monitoring can offer benefit in the prevention of severe hyperglycemia and hypoglycemia because trends in BG concentrations can be more readily identified.^33^ Many studies have investigated whether the accuracy of the continuous glucose monitoring devices was compromised in patients suffering from given pathological conditions (eg, septic shock, acute renal failure) or receiving specific drugs (eg, vasoactive drugs).^34^, ^35^ To our knowledge, no studies in the human medical literature have specifically investigated the influence of changes in metabolic variables during DKA on the accuracy of devices measuring IG. However, results from studies investigating CGMS performance in intensive care patients (suffering from pathological conditions other than DKA) have indicated that low pH, high lactate concentrations, and the use of vasoactive drugs do not compromise agreement between BG and IG measurements.^36^, ^37^, ^38^

To investigate if specific patient factors or metabolic variables could account for the difference in accuracy of the FGMS, we evaluated the effects of BCS, time wearing, lactate concentration, and severity of ketosis and acidosis in each subject. No variable was found to influence the agreement between IG and BG results. A prospective study in veterinary medicine that evaluated the effects of hydration, BCS, measures of perfusion (Doppler blood pressure, lactate and rectal‐axillary temperature difference), and severity of ketosis on the performance of a CGMS (CGMS Gold, Medtronic Minimed, California) in dogs and cats with DKA found only a weak association between hydration and the accuracy of the measurements, with the device being more accurate in more hydrated patients.^39^ Results of this study suggest that this device is a clinically useful tool for monitoring IG concentration in critically ill patients, but it has a number of disadvantages, including the initial cost of the device, the cost of the sensor, and the need to obtain blood samples for calibrations every 8‐12 hours.^39^ The most important limitation is that glucose measurements are only available retrospectively, after downloading the data onto a personal computer, thereby limiting their clinical usefulness in the management of hospitalized patients.^39^ A more recent generation of CGMS (Guardian REAL‐Time continuous glucose monitoring system, Medtronic, Münchenbuchsee, Switzerland) provides IG concentrations in real time, enables onscreen data recording over a 24‐hour period, and can be left in place for up to 72 hours. Its accuracy has been investigated in dogs and cats,^20^, ^40^, ^41^, ^42^ but a direct comparison between the performances of this CGMS device and the Freestyle Libre investigated in our study is not possible because of the different accuracy criteria used. Despite its advantages, this device still has the limitation of requiring calibration at least twice daily. The FGMS overcomes these limitations by providing IG concentrations in real time and is factory‐calibrated without requiring additional BG measurements for calibration.

In our study, the correlations between IG concentrations measured by the FGMS and BG concentrations obtained by the PBGM before and after DKA resolution were *r* = .88 and *r* = .93, respectively. Our results, especially those obtained after DKA resolution, compare favorably with an earlier veterinary study of the accuracy of the FGMS in stable diabetic dogs, which found a similar correlation with peripheral BG concentrations measured by the hexokinase method (*r* = .94).^21^

Before DKA resolution, the percentages of results within ±15 mg/dL of the BG concentration for BG concentrations <100 mg/dL and within ±15% of the BG concentration for BG concentrations ≥100 mg/dL were 0% and 43.8%, respectively, and therefore analytical accuracy, based on ISO 15197:2013 requirements, was not obtained. After DKA resolution, the percentages improved to 42.9% and 53.6% for BG concentrations <100 mg/dL and ≥100 mg/dL, respectively. Therefore, even after metabolic abnormalities were resolved, the ISO analytical standards were not fulfilled. Although their results also did not meet ISO 15197:2013 requirements, a previous study found better results in stable diabetic dogs, with 56% of FGMS measurements within ±15 mg/dL of the BG concentration for BG concentrations <100 mg/dL and 73% within ±15% of the BG concentration for BG concentrations ≥100 mg/dL.^21^ These differences may depend on the fact that the previous study^21^ evaluated the performance of the FGMS by comparing it with the hexokinase method and not with a PBGM. Parkes EGA showed acceptable clinical accuracy both before and after DKA resolution, with 100% and 99.6%, respectively, of the FGMS readings in zones A and B, similar to reported rates of 99.5% in a study of humans using the FGMS to monitor critically ill patients with diabetes,^35^ and better than the reported rates of 98.7% in the previous veterinary study evaluating the use of the FGMS in non‐DKA diabetic dogs.^21^

The ISO 15197:2013 standards require comparison of BG meter measurements with the results of a standard reference method. However, these standards are designed for comparisons between results from a single compartment, typically the blood, and comparisons between 2 different compartments (blood and interstitial fluid) may be inappropriate, because of physiological difference between these compartments. In the absence of established standard criteria for evaluation of the accuracy of continuous glucose measurements in the interstitial fluid, the ISO criteria for the evaluation of PBGMs provide a relevant substitute to identify devices that are as close as possible to meeting accuracy criteria and that are not dangerous for the animal's health. Currently, studies in human and veterinary literature adopt this approach. With this caveat, the FGMS can be considered acceptable for clinical use, despite the analytical accuracy requirements not being met.

Figure 3 shows that there was significant inter‐patient variability in the accuracy of the FGMS, as observed in studies evaluating CGMS in stable diabetic dogs, and in dogs and cats with DKA.^16^, ^39^ Given the marked inter‐patient variability, we strongly recommend checking BG concentration in patients whenever unexpected or low FGMS results are obtained.

In our study, 1 dog developed mild erythema at the site of the sensor, which could be related to the patch used to ensure adhesion of the device to the cutaneous surface. However, an allergic contact sensitization caused by the device cannot be ruled out.^43^ In a study of humans, mild dermatological signs such as pruritus, erythema, edema, rash, induration, bruising, and bleeding were observed in <9% of cases.^44^

Our study had some limitations. Capillary and venous BG concentrations, obtained using a human PBGM validated for use in dogs^25^ and not using the classical reference method (hexokinase), were used as a reference to evaluate the accuracy of the FGMS. FreeStyle Libre is an IG monitoring system intended to be a replacement for PBGMs, and therefore capillary and venous BG concentrations obtained using a PBGM may be considered an appropriate comparator in evaluating the performance and accuracy of this factory‐calibrated system. However, it is known that BG concentrations determined by most PBGM devices designed for use in human diabetic patients typically are lower than actual BG concentrations determined by reference methods, and this difference increases as hyperglycemia worsens.^45^ Therefore, it is plausible that the measures of the average difference between IG and BG concentrations could be better or worse depending on the reference glucose measurement chosen. A further limitation was the limited number of data points in the hypoglycemic range; only 26 BG concentrations obtained with the PBGM were <100 mg/dL (5 obtained before DKA resolution and 21 after DKA resolution), and this number is not sufficient to argue that accuracy in the hypoglycemic range was adequately evaluated.

Another limitation is that the skin and SC adipose tissue thickness at the site of application of the sensor were not evaluated, making it impossible to assess their influence on the accuracy of the FGMS. In human medicine, tissue glucose concentration nadirs in muscle have been reported to be delayed in time and lower in magnitude relative to glucose concentrations in adipose tissue and blood, especially during insulin‐induced hypoglycemia.^46^, ^47^ Decreased thickness of the SC adipose tissue layer may result in closer sensor proximity to the underlying muscle tissue and, consequently, in inaccurate glucose concentration results.

Other limitations of our study include the use of a single sensor for each dog, such that the precision of the FGMS was not investigated. In veterinary medicine, 2 studies investigated the effect of sensor location on performance of the Guardian REAL‐Time CGMS.^41^, ^42^ In cats, preliminary results suggest that dorsal neck placement may be superior to lateral chest wall and lateral knee fold placement.^41^ In dogs, IG concentrations obtained by the CGMS at the lateral thorax site had the best correlation with BG concentrations compared to lateral neck, lumbar, and abdomen sites.^42^ In our study, the sensor was placed at a single body site (the dorsal part of the neck, an area not particularly subject to traction and trauma, especially in animals in lateral recumbency), not allowing evaluation of the application site as a variable that might influence the accuracy of the device.

In conclusion, although the ISO 15197:2013 requirements were not fulfilled, the novel FGMS provides clinically accurate estimates of BG concentration compared with PBGM and represents a useful device to monitor BG concentration in critically ill hospitalized dogs with DKA. Acid‐base status, BHB and lactate concentrations, BCS, and time wearing did not influence the accuracy of the sensor, making it suitable not only for stable diabetic dogs but also for dogs with DKA.

## CONFLICT OF INTEREST DECLARATION

Authors declare no conflict of interest.

## OFF‐LABEL ANTIMICROBIAL DECLARATION

Authors declare no off‐label use of antimicrobials.

## INSTITUTIONAL ANIMAL CARE AND USE COMMITTEE (IACUC) OR OTHER APPROVAL DECLARATION

The Scientific Ethics Committee of the University of Bologna (Italy) approved this study.

## HUMAN ETHICS APPROVAL DECLARATION

Authors declare human ethics approval was not needed for this study.
